# One-step preparation of a novel SrCO_3_/g-C_3_N_4_ nano-composite and its application in selective adsorption of crystal violet

**DOI:** 10.1039/c7ra11565b

**Published:** 2018-02-07

**Authors:** Peng Lu, Xueli Hu, Yujie Li, Meng Zhang, Xiaoping Liu, Youzhou He, Fan Dong, Min Fu, Zhi Zhang

**Affiliations:** College of Urban Construction and Environmental Engineering, Chongqing University Chongqing 400045 China zhangzhicqu2016@126.com; College of Environment and Resources, Chongqing Technology and Business University, Chongqing Key Laboratory of Catalysis and New Environmental Materials Chongqing 400067 China

## Abstract

A novel kind of nanoparticle SrCO_3_/g-C_3_N_4_ was prepared using strontium carbonate (SrCO_3_) and melamine (C_3_H_6_N_6_) as raw materials *via* one-step calcination. The formation of SrCO_3_/g-C_3_N_4_ was confirmed from the X-ray diffraction (XRD), Fourier transform infrared spectra (FT-IR), Scanning Electron Microscopy (SEM), Transmission Electron Microscopy (TEM), Brunauer–Emmett–Teller (BET) and X-ray photoelectron spectroscopy (XPS) analysis. Its selective adsorption performance was evaluated towards crystal violet (CV), rhodamine B (RhB) and methylene blue (MB). The results showed that the SrCO_3_/g-C_3_N_4_ had selective adsorption ability of CV. Furthermore, adsorption measurements of CV were conducted to investigate the influences of contact time, initial concentration, initial dye solution pH value and adsorbent dosage. The maximum removal rate of CV was 98.56% when the initial concentration was 1600 mg L^−1^. The kinetic study indicated the adsorption of CV followed the pseudo-second-second model well. The adsorption efficiency of SrCO_3_/g-C_3_N_4_ was greater (97.46%) than that of g-C_3_N_4_ (31.30%) and SrCO_3_ (17.30%). It could be deduced that the synergistic effect of conjugation interaction of g-C_3_N_4_ and the electrostatic attraction of SrCO_3_ might be the main driving force for the superb adsorption of CV.

## Abstract 2

A novel kind of nanoparticle SrCO_3_/g-C_3_N_4_ was prepared using strontium carbonate (SrCO_3_) and melamine as raw materials *via* one-step calcination.

## Graphical abstract

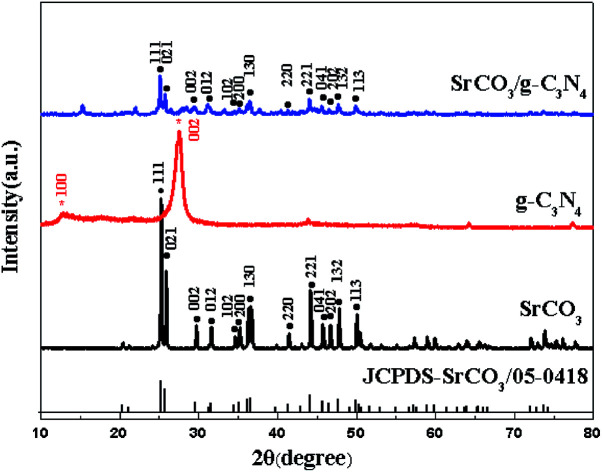

## 1 Introduction

Nowadays, graphitic carbon nitride (g-C_3_N_4_) has gained considerable research attention because it possesses excellent advantages such as high chemical and thermal stability under ambient temperature, cost-effective, non-toxic and simple preparation.^1,2^ g-C_3_N_4_, as a new intriguing class of graphite analogue, consists of conjugated planes containing highly ordered tri-*s*-triazine (C_6_N_7_) units. The layered structure of the tri-*s*-triazine is connected by weak forces-van der Waal forces. It has been widely used in the photocatalysis field for it has an energy band gap of 2.7 eV which makes it is capable of the visible adsorption.^3–6^ As a photocatalyst, it has been widely used in water splitting,^7,8^ organic pollutants degradation,^9,10^ CO_2_ reduction,^11^ and other fields.^12,13^

Crystal violet (CV, C_25_H_30_ClN_3_, IUPAC name is *N*-[4-[bis][4-dimethyl-amino]-phenyl]methylene]-2,5-cyclohexadien-1-ylidine]-*n*-methylmethanaminium chloride), a typical triphenylmethane dye, is widely used in cell biology, paper, leather and textile industry.^14–16^ The wastewater containing CV is low biodegradability and high stability (complex aromatic structure) and it could be absorbed through the skin and causing the skin, eye, digestive irritation and even cancer.^17–19^ From the aspect of environmental safety and health of life, it is vital to develop an effective way to abate CV in wastewater. Various methods have been adopted for eliminating dye pollution from water, including chemical oxidation,^20,21^ photo-catalytic decomposition,^22,23^ electro-catalytic degradation^24,25^ and non-thermal plasma.^26–28^ These methods usually have some defects, such as slow degradation rate, complex, heavy expenses and usually causing secondary pollution. The adsorption technique is gaining more attention for it is high efficiency, simple design, cost-effective and adaptable.^29–35^ There have been various kinds of adsorbents developed, such as carbon materials,^36–38^ natural clay minerals,^39–42^ and bioadsorbents.^43–45^ However, certain deficiencies including costly, intricate pre-treatment and low adsorption capability limited the use of some certain adsorbents.^46–53^

Due to the conjugated region, stacking structure and the weak forces of g-C_3_N_4_, it has the potential for aggregating functional groups or materials to form nanocomposite with multiform favorable properties.^54–56^ This distinctive structure has drawn great interesting in improving the photocatalytic performance.^57,58^ But the research of using modified g-C_3_N_4_ as adsorbent for clearing up dye pollutant is rarely been reported.^59,60^ As far as we know, there has been no report of using g-C_3_N_4_-based composite as an adsorbent in dye wastewater treatment. Strontium carbonate (SrCO_3_), a typical alkaline earth metal carbonate, has been widely used as additives in industrial production.^61–63^ Meanwhile, some research has been reported on its adsorption performance attributed to its various architectures.^64,65^

In this work, we firstly induced SrCO_3_ to incorporate with g-C_3_N_4_*via* one-step calcination method to fabricate a novel adsorbent SrCO_3_/g-C_3_N_4_. The morphology and structure of the composite was characterized by XRD, FT-IR, SEM, TEM, BET and XPS, and its adsorptive capacity and selectivity of CV in aqueous solution were investigated. To the best of our knowledge, this work represents the first example employing g-C_3_N_4_-based composite for selective adsorption of CV.

## 2 Experimental

### 2.1 Synthesis of SrCO_3_/g-C_3_N_4_

SrCO_3_, melamine and CV are all AR grade and purchased from Chengdu Kelong Chemical Agents (China), without any further purification. SrCO_3_/g-C_3_N_4_ was synthesized by one-step calcination process in a muffle furnace. SrCO_3_ and melamine (mass ratio = 1 : 1) were dissolved with deionized water in alumina crucible, and then the mixed solution was dispersion with ultrasonic irradiation for 20 min under ambient temperature. The final solution was transferred into muffle furnace and maintained at 600 °C for 4 hours at heating rate of 5 °C min^−1^ to obtain the prepared nanocomposite. SrCO_3_ and melamine were also treated as the forward route for making comparison.

### 2.2 Characterization

X-ray diffraction (XRD, Shimadzu, XRD-6100) patterns were detected with Cu Kα radiation (40 kV, 30 mA, 2*θ* = 10–80°) to investigate the crystal structures of the samples. Fourier transform infrared spectra (FT-IR, Shimadzu, IR Prestige-21), were recorded in the range of 4500–400 cm^−1^, using KBr technique to analyze the functional groups on the surface of the composite. Scanning electron microscope (SEM, JEOL, JSM-7800F) and Transmission Electron Microscopy (TEM, JEOL, JEM-2100) were used to observe the morphologies and the microstructures of the samples. The N_2_ adsorption apparatus (Micromeritics, ASAP 2020) were used to obtain the Brunauer–Emmett–Teller (BET) surface area of the samples. X-ray photoelectron spectroscopy (XPS, Thermo Scientific, ESCALAB 250xi) was used to determine the binding energy.

### 2.3 Adsorption experiments

The concentration of CV in the solution was determined at the maximum absorbance (*λ*_max_ = 580 nm) by UV-vis spectrophotometer (UV-vis spectrometer, Tianmei, UV1102). For high concentration, the samples were diluted before measurements. And the initial pH of the dye solution was measured by pH meter (pH meter, Sartorius, PB10).

The adsorption experiments were conducted by a batch method. All experiments were conducted at 7.0 pH value, except those that investigated the effect of initial pH of dye solution. And 0.08 g adsorbent was dispersed in 80 mL CV solution, except that were used to study the effect of the dosage. Similarly, the initial concentration is 1600 mg L^−1^, except those that investigate the same parameters. The pH of the initial dye solution was adjusted with HCL (0.1 mol L^−1^) and NaOH (0.1 mol L^−1^). The kinetic experiments were carried out at the initial concentration of 500, 1000, 1200, 1400, and 1600 mg L^−1^. It performed on an air bathed shaker. The solution was separated by centrifugation at 5000 rpm for 5 min.

The adsorption capacity at time *t q*_*t*_ (mg g^−1^) and removal rate (%) were calculated using the following equation:1
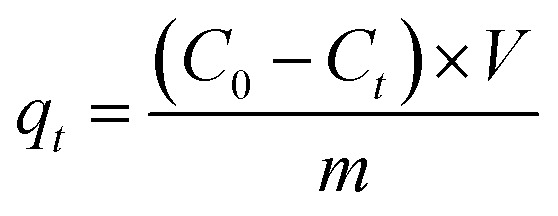
2
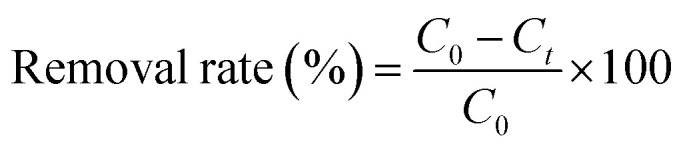
where *C*_0_ and *C*_*t*_ are the liquid-phase concentrations of the CV (mg L^−1^) at initial time and time *t*, respectively. *V* is the volume of the solution (mL) and *m* is the mass of the used adsorbent (mg).

## 3 Results and discussion

### 3.1 Characterization of the samples

**Fig. 1:**
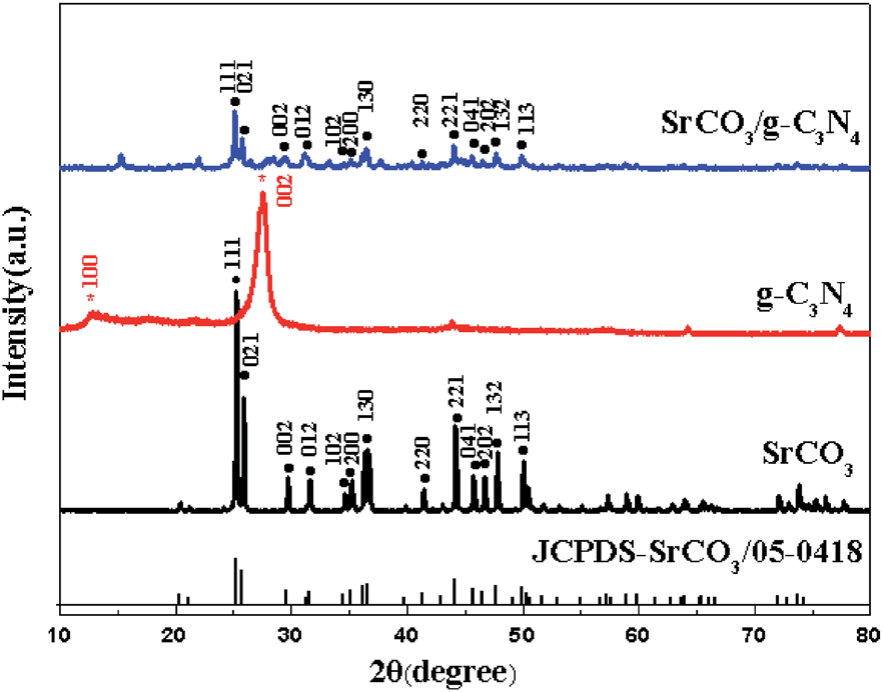
XRD patterns of the g-C_3_N_4_, SrCO_3_ and SrCO_3_/g-C_3_N_4_.

The XRD patterns of the prepared samples are shown in Fig. 1. The g-C_3_N_4_ displayed a typical diffraction peaks at 27.46° and 12.96°, corresponding to the (002) and (100) diffraction planes, which represented the interlayer stacking reflection and in-plane structure of aromatic system, respectively.^66,67^ This indicated that the g-C_3_N_4_ was synthesized by polycondensation approach with pure melamine. Meanwhile, the observed diffraction peaks of calcined SrCO_3_ are located at 2*θ* angles = 25.28°, 25.91°, 29.73°, 31.62°, 34.64°, 35.22°, 36.64°, 41.42°, 44.18°, 45.74°, 46.68°, 47.80°, 50.03° which correspond to the planes of (111), (021), (002), (012), (102), (200), (130), (220), (221), (041), (202), (132), (113), respectively. It could be unambiguously indexed to the orthorhombic phase of SrCO_3_ (JCPDS card no. 05-0418).^68,69^ There is no observation of the typical diffraction of g-C_3_N_4_ in the synthesized composite, the diffraction peaks were mostly the same as the SrCO_3_, but the feature peaks positions of SrCO_3_ shifted slightly toward a lower diffraction angle. And the impure peaks of the composite might be the carbonization of the raw materials or the melamine did not condense completely.

**Fig. 2:**
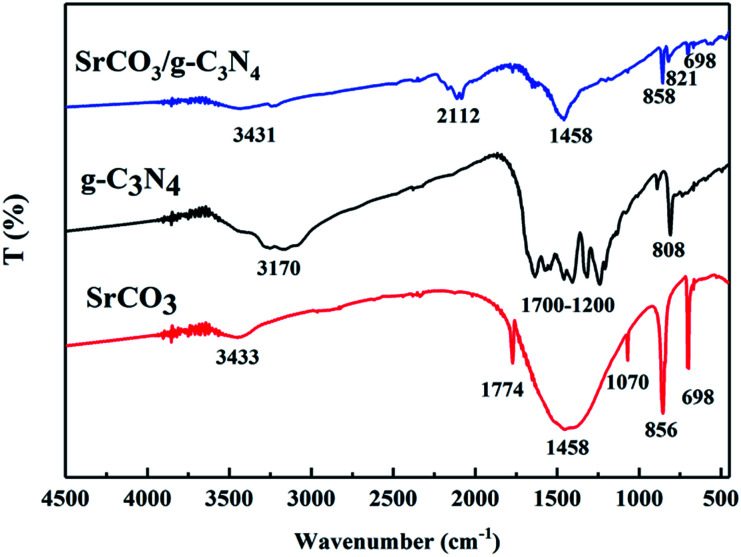
FT-IR spectra of the g-C_3_N_4_, SrCO_3_ and SrCO_3_/g-C_3_N_4_.

The FT-IR spectra of the prepared materials are shown in Fig. 2. For the calcined melamine, the typical adsorption peaks in the 1200–1700 cm^−1^ range and at 808 cm^−1^ of g-C_3_N_4_ could be observed. They are assigned to the typical skeletal stretching vibrations of armomatic C–N heterocycles and the out-of-plane bending vibration of tri-*s*-triazine rings, respectively.^70,71^ The peaks at 698, 858, 1070, 1458 and 1774 cm^−1^ corresponding to the CO_3_^2−^ of the calcined SrCO_3_ were observed.^50^ In case of the SrCO_3_/g-C_3_N_4_, the typical skeletal stretching vibrations of tri-*s*-triazine were hardly observed; it might be the adsorption intense of SrCO_3_ was so strong in order to impede the peaks of g-C_3_N_4_. Meanwhile, the peak at 808 cm^−1^ presented in g-C_3_N_4_ shifted to 821 cm^−1^, the peak at 856 cm^−1^ presented in SrCO_3_ shifted to 858 cm^−1^, the peaks at 1070 cm^−1^ and 1774 cm^−1^ were disappeared, these might due to the strong interactions between the carbonate of SrCO_3_ and tri-*s*-triazine rings of g-C_3_N_4_. Furthermore, the new peak at 2112 cm^−1^ corresponding to C≡N groups appeared after calcination indicating that the incorporation of SrCO_3_ has intense interaction during the condense process of melamine.^72^

**Fig. 3:**
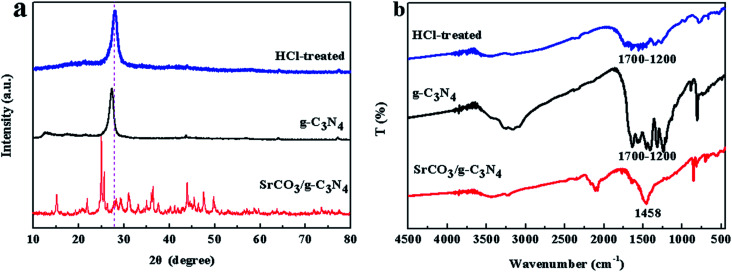
XRD patterns (a) and FT-IR spectra (b) of the HCl-treated SrCO_3_/g-C_3_N_4_.

To make further investigation of the constitution of the prepared composite, the calcined product was treated with HCl (0.5 mol L^−1^) and deionized water, because g-C_3_N_4_ possessed fascinating acid stability. The treatment was terminated until the pH value was neutral, and then the treated nanocomposite was dried at 60 °C for 24 h. The XRD patterns and FT-IR spectra of the HCl-treated product are characterized in Fig. 3. The feature diffraction peak of g-C_3_N_4_, indicating the interlayer stacking, changed from 27.46° to 27.92° is explicitly observed in Fig. 3a. The change of the diffraction peak was corresponded to the stacking distance reduced from 0.325 to 0.319 nm. The above results implied that the interlayer stacking order was improved. And FT-IR spectra of the product were well matched with the pure g-C_3_N_4_ except the weak intensity (Fig. 3b). So, it could be indicated that the SrCO_3_/g-C_3_N_4_ was formed with the raw materials SrCO_3_ and melamine.

**Fig. 4:**
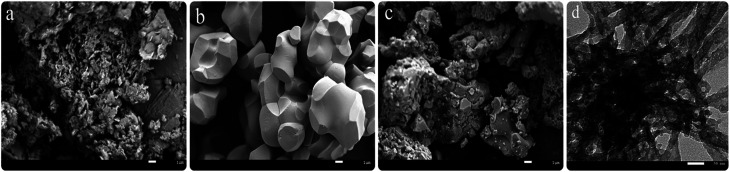
SEM images of the g-C_3_N_4_ (a), SrCO_3_ (b) and SrCO_3_/g-C_3_N_4_ (c); TEM images of the SrCO_3_/g-C_3_N_4_ (d).

SEM and TEM images of the calcined samples are displayed in Fig. 4. It revealed the morphology and microstructure of SrCO_3_/g-C_3_N_4_. In Fig. 4a, it could be seen the calcined melamine was predominantly composed of plate-like sheets,^73^ and the morphology of calcined SrCO_3_ was irregular polyhedrons with smooth surface is shown in Fig. 4b. In Fig. 4c, it could be seen clearly that the g-C_3_N_4_ covered on the SrCO_3_ incompletely. Further structural details of SrCO_3_/g-C_3_N_4_ are shown in Fig. 4d. There were some step edges of the layered g-C_3_N_4_ and rods-like SrCO_3_ with some parts overlapping. It might be the amount of g-C_3_N_4_ was not enough to disperse on the entire surface of SrCO_3_; and some areas cannot be wrapped by the g-C_3_N_4_. The SEM and TEM image could reveal that there existed some interactions between these two materials. Meanwhile, the specific surface areas of the clained samples were measured. As shown in Table 1, compared with g-C_3_N_4_ the composite BET surface area gets smaller with the addition of SrCO_3_, this might be the SrCO_3_ interacted with the stacking structure of g-C_3_N_4_. These results consistent with SEM results which could further prove that there have been some interactions between SrCO_3_ and g-C_3_N_4_.

**Table 1:** Textural properties of g-C_3_N_4_, SrCO_3_ and SrCO_3_/g-C_3_N_4_^a^

Sample	*S*_BET_ (m^2^ g^−1^)	*V*_total_ (cm^3^ g^−1^)	*D* (nm)
g-C_3_N_4_	27.4	0.126	17.5
SrCO_3_	1.93	0.003	8.72
SrCO_3_/g-C_3_N_4_	5.86	0.016	9.69

a *S*_BET_: BET surface area, *V*_total_: total pore volume, *D*: average pore diameter calculated using BJH method.

**Fig. 5:**
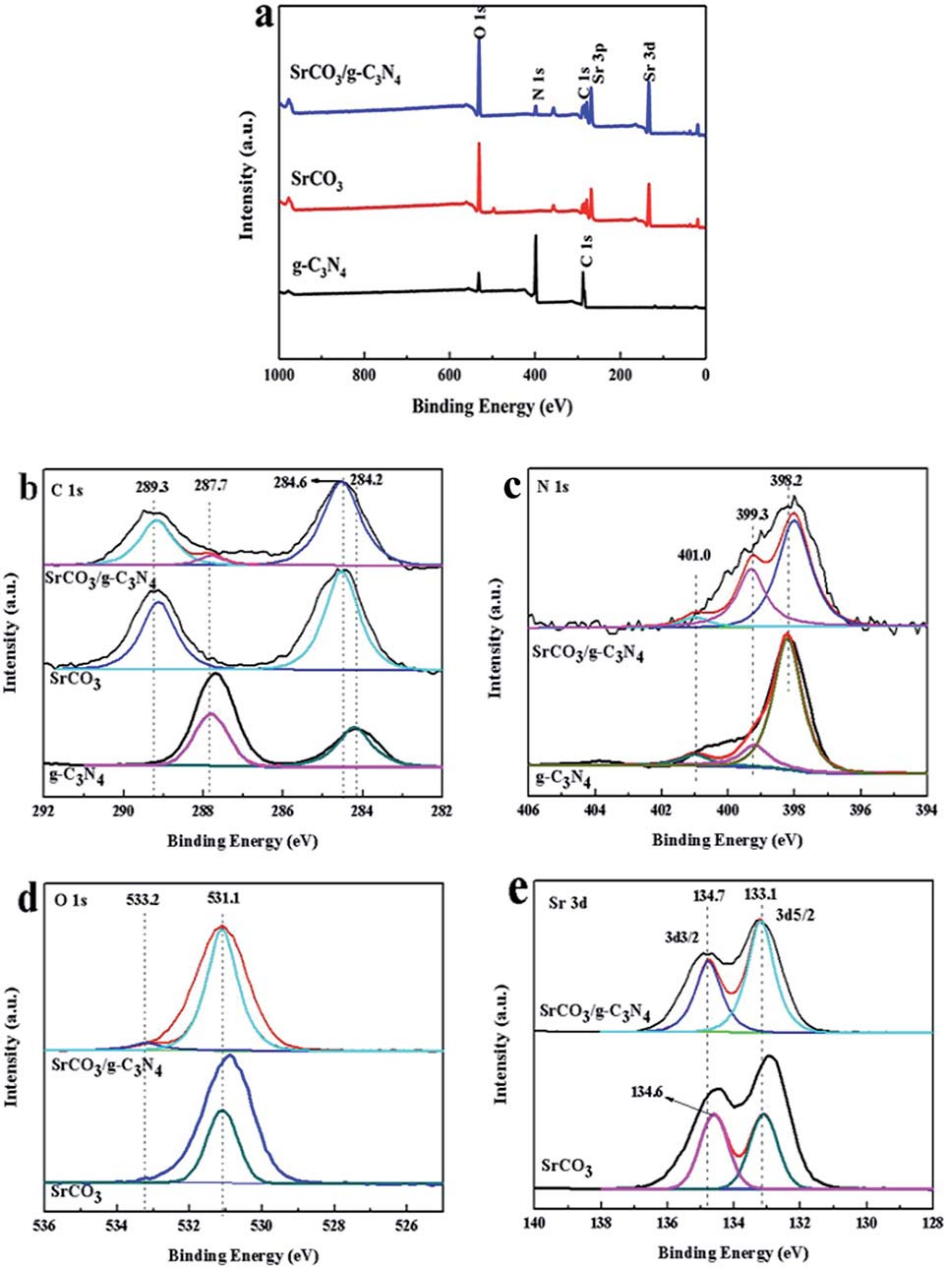
XPS survey spectra of g-C_3_N_4_, SrCO_3_ and SrCO_3_/g-C_3_N_4_ (a) and high-resolution XPS spectrum: C 1s (b), N 1s (c), O 1s (d) and Sr 3d (e).

To further study the chemical state of SrCO_3_/g-C_3_N_4_, the XPS measurements were carried out. Fig. 5a is the general XPS spectra of calcined materials, indicating the presence of C, N, O and Sr in SrCO_3_/g-C_3_N_4_. The XPS spectra of C 1s are shown in Fig. 5b. For pristine g-C_3_N_4_ the peaks at 284.2 eV, 287.7 eV were corresponding to sp^2^-hybridized carbon in C–C group or the adventitious hydrocarbon^74^ and sp^2^-bond carbon in form of C–C=N.^75^ But after the blended calcinations, the peak at 284.2 eV shifted to 284.6 eV. The N 1s spectra are shown in Fig. 5c. The peak at 398.2 eV was attributed to sp^2^-hybridized nitrogen in N atom aromatic rings in form of C–N=C,^76^ and the nearly peaks at 399.3 eV and 401.0 eV were regarded as tertiary nitrogen (N-(C)_3_) and amino functional groups, respectively.^77^ After incorporation, the binding energy peaks slightly shifted. The peaks at 531.1 eV and 533.2 eV of O 1s spectra corresponding to the SrCO_3_ have no significant change in Fig. 5d. The XPS spectra of Sr 3d at 133.1 eV and 134.6 eV for calcined SrCO_3_ were observed in Fig. 5e. These peaks was corresponding to the Sr 3d5/2 and Sr 3d3/2, and the peak at 134.6 eV shifted to 134.7 eV after calcination. All these changes after co-calcination suggested that there were strong interactions between the two raw materials. Therefore, according to the analysis of XPS and the above results of XRD, FT-IR, SEM, TEM and BET measurements, it could be concluded that the SrCO_3_/g-C_3_N_4_ was synthesized using SrCO_3_ and melamine by one-step calcination.

### 3.2 The selective adsorption of CV

In our previous study, cationic dyes MB, RhB and CV were chosen as the target dye to conduct the adsorption experiments. We found that SrCO_3_/g-C_3_N_4_ appeared efficient adsorption capacity for CV except for the other dye solutions. To make further investigation of the selectivity of SrCO_3_/g-C_3_N_4_, mixed solutions of CV/MB, CV/RhB and CV/MB/RhB with equal volume ratio were prepared. The concentration of each dye solution was 10 mg L^−1^, and dosage ratio of SrCO_3_/g-C_3_N_4_ and mixed solutions was 1 g L^−1^. The adsorption experiments were carried out under ambient temperature and different pH value for 120 min.

**Fig. 6:**
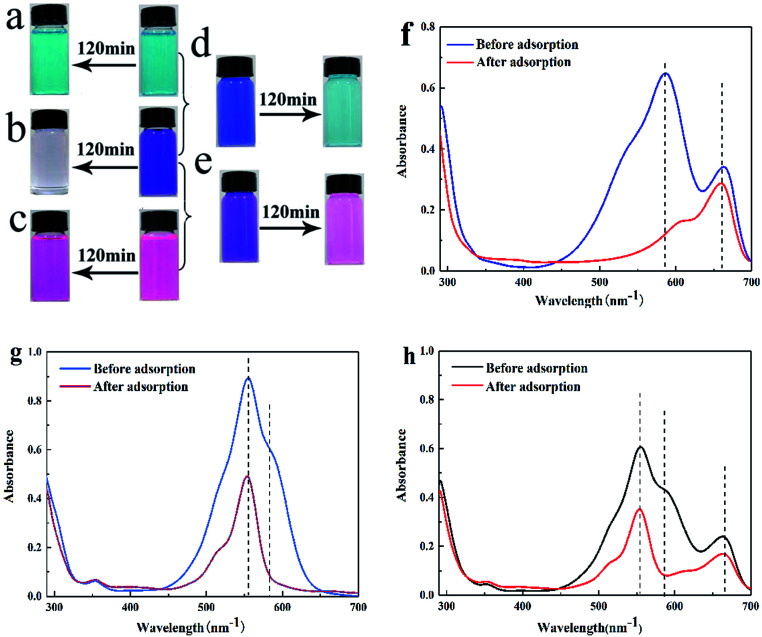
Optical photographs of single adsorption of MB (a), CV (b) and RhB (c); the selective adsorption of CV from CV/MB (d) and CV/RhB (e) mixture, UV-vis spectra of CV/MB (f),CV/RhB (g) and CV/MB/RhB (h) mixture before and after adsorption.

The optical pictures and UV-vis measurements of the adsorption process are shown in Fig. 6. It could be apparently seen that CV was decolorized after the test (Fig. 6b), but in the MB and RhB groups the color slightly changed (Fig. 6a–c). In Fig. 6d, the color of CV/MB mixed solution was changed from dark blue to incipient blue (the color corresponding to the MB). For the CV/RhB group, the end color is very close to RhB (Fig. 6e). It suggested that CV in these two mixtures was selectively captured. However, the color in group CV/MB/RhB did not change, because the color of MB and RhB mixed solution is the same as that of CV. To further investigate the selective adsorption of CV, the UV-vis spectra were conducted for the tested groups. In the case of CV/MB, the UV-vis adsorption peaks of CV and MB were at 580 nm and 664 nm before adsorption, respectively. After adsorption, the peak of CV was dropped down drastically while the MB peak was almost unchanged (Fig. 6f). The same situation happened in the CV/RhB group (Fig. 6g). Although the optical of CV/MB/RhB group did not change, but the UV-vis adsorption curve appeared the same situation with the other two mixed groups (Fig. 6h). It could be concluded that the SrCO_3_/g-C_3_N_4_ performed selective adsorption of CV from CV/MB/RhB.

**Fig. 7:**
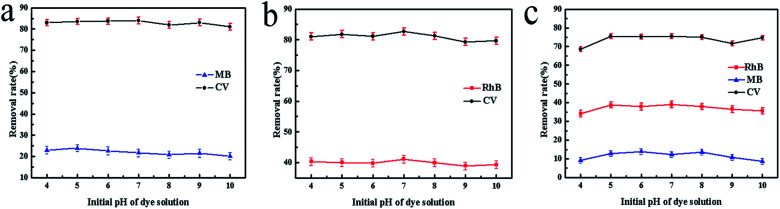
Effect of the initial pH of mixed dye solution CV/MB (a), CV/RhB (b) and CV/MB/RhB (c).

Furthermore, we also evaluated the effect of the initial pH of the dye solution. Fig. 7 shows the tested results. It could be seen that the initial pH (ranged from 4 to 10) of mixed dye solution did not significantly influence the adsorption process in each tested group.

#### 3.2.1 Effect of contact time

**Fig. 8:**
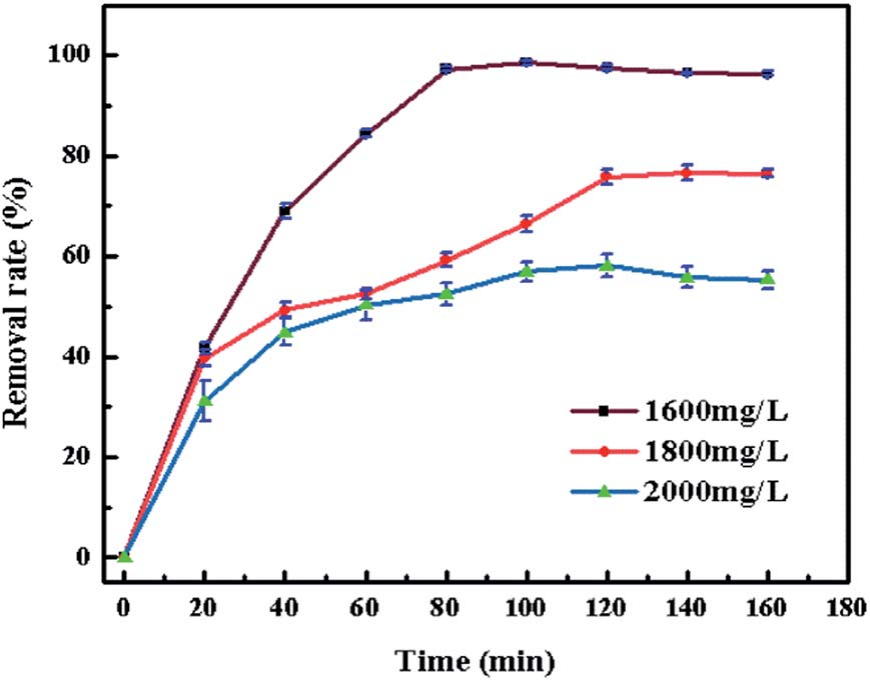
Effect of contact time.

Fig. 8 shows the effect of different contact time (0–160 min) in the adsorption process. The initial concentration of CV is 1600, 1800 and 2000 mg L^−1^ and the dosage ratio of SrCO_3_/g-C_3_N_4_ and CV solution was 1 g L^−1^. For all concentrations, the removal rate of CV became constant after 120 min. Meanwhile, the group of 1600 mg L^−1^ reached the equilibrium firstly and the removal rate was up to the other concentration. So, the contact time of 120 min and the initial concentration of CV of 1600 mg L^−1^ were chosen for the following investigation.

#### 3.2.2 Effect of initial concentration of CV

**Fig. 9:**
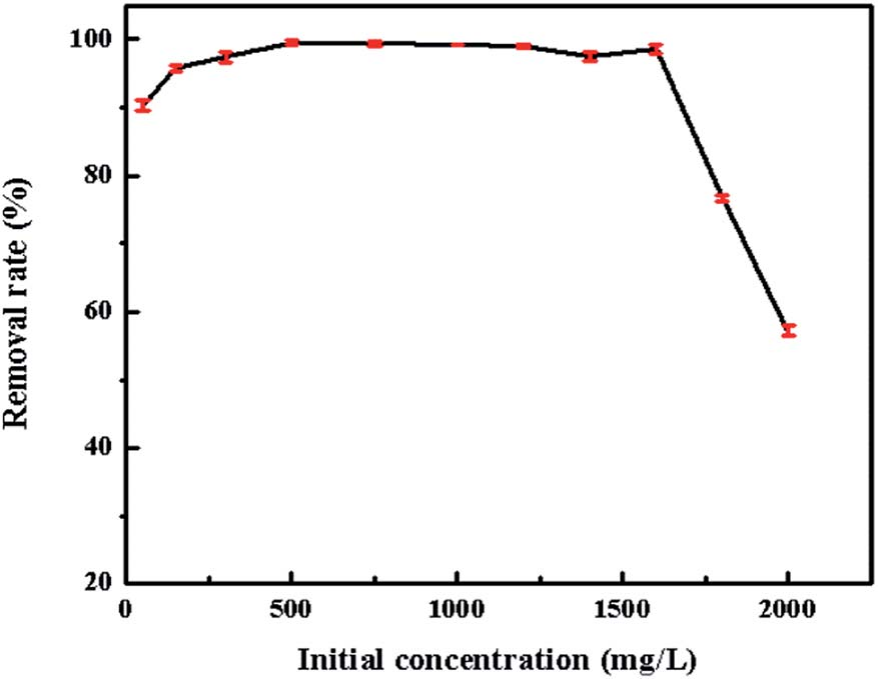
Effect of initial CV concentration.

The effect of initial concentration (50–2000 mg L^−1^) of CV is shown in Fig. 9. From the obtained results, it was found that the removal efficiency kept steadily before the initial dye concentration up to 1800 mg L^−1^. This might be attributed to the higher initial concentration offered more numbers of dye molecules that could contact easily with SrCO_3_/g-C_3_N_4_. However, the removal rate was reduced at higher initial concentration indicating the saturation of SrCO_3_/g-C_3_N_4_.

#### 3.2.3 Effect of adsorbent dosage

**Fig. 10:**
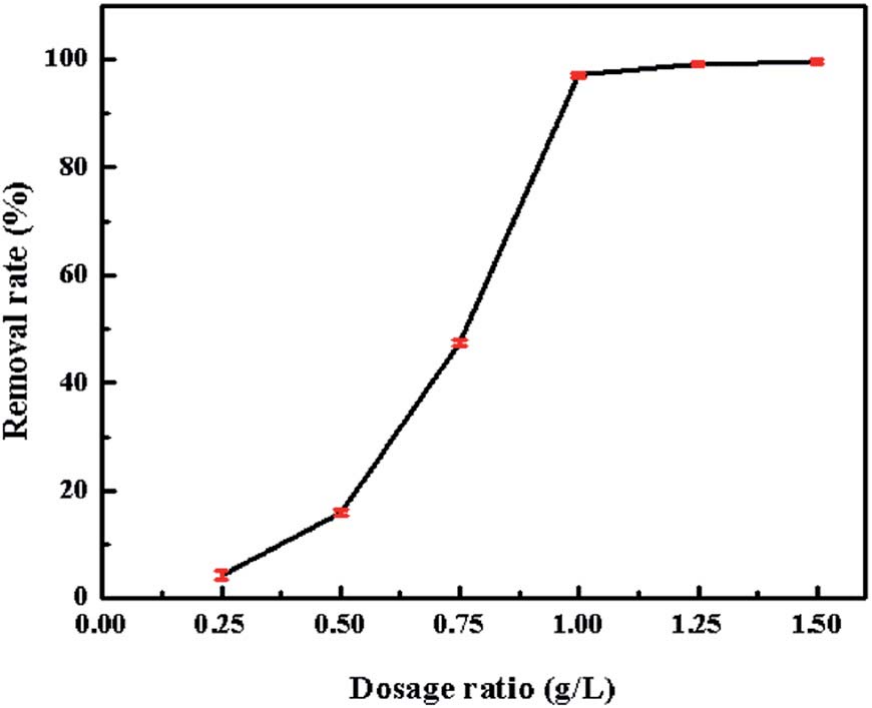
Effect of SrCO_3_/g-C_3_N_4_ dosage.

The dosage of the adsorbents is one of the major parameter which influences the adsorption process; an appropriate amount of adsorbents is in favor of the real industrial treatment. Fig. 10 shows the effect of the adsorbent dosage (0.25, 0.50, 0.75, 1.00, 1.25, 1.50 g L^−1^) of the CV. The removal rate increased with the increased amount of the adsorbent. When the dosage ups to 1.00 g L^−1^, the removal efficiency did not increase with the increasing dosage. It might due to the binding of almost CV ions onto SrCO_3_/g-C_3_N_4_, which made the equilibrium between solution and adsorbents. So the amount of adsorbent 1.00 g L^−1^ was the suitable dosage for the adsorption of CV.

#### 3.2.4 Effect of pH

**Fig. 11:**
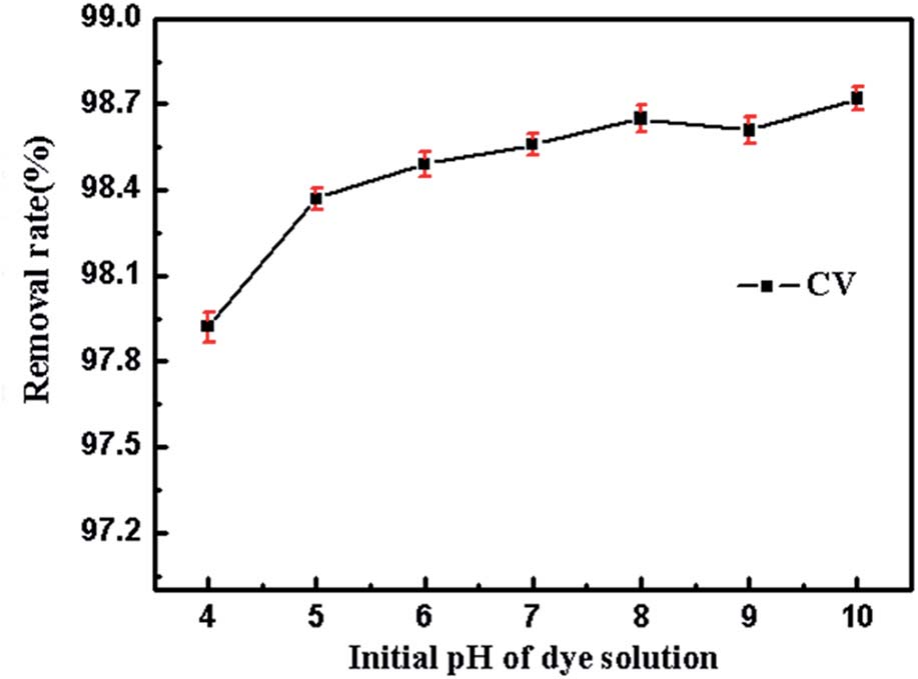
Effect of the initial pH of CV.

The pH of initial dye solution is a key factor in adsorption process, which could influence the interaction between adsorbent and dye. In order to find out the pH effect, the adsorption test was conducted at initial solution pH ranged from 4 to 10. The results in Fig. 11 show that the initial pH of CV solution had little effect on the adsorption capacity. This observation could demonstrate that SrCO_3_/g-C_3_N_4_ is suitable for removing CV in wastewater in a wide pH range, and there is no need to adjust the initial solution pH before treatment.

### 3.3 Adsorption kinetics

In order to study the characteristics of the adsorption process, pseudo-first-order and pseudo-second-order kinetics models were carried out with different concentration (500, 1000, 1200, 1600 mg L^−1^). The pseudo-first-order and pseudo-second-order are expressed by eqn (3) and (4).^78^

3 ln(*q*_*e*_ − *q*_*t*_) = ln *q*_e_ − *k*_1_*t*

4
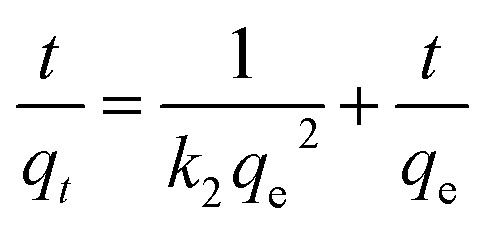
where *k*_1_ (min^−1^) and *k*_2_ (g (mg^−1^ min^−1^)) are the rate constant of pseudo-first-order and pseudo-second-order model, respectively; *q*_e_ (mg g^−1^) and *q*_*t*_ (mg g^−1^) are the adsorbed amount at equilibrium time and time *t*.

**Fig. 12:**
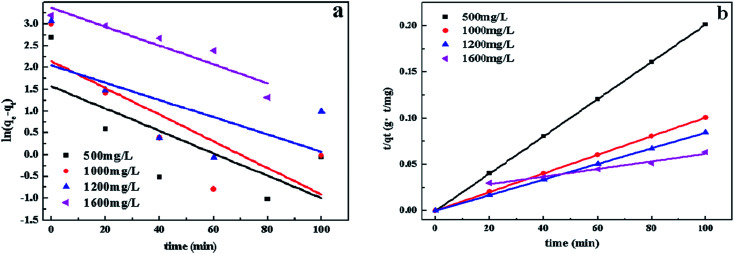
Pseudo-first-order (a), and pseudo-second-order (b) kinetic plots for adsorption.

The rate constant of adsorption could be calculated from the slope of the plot in Fig. 12 and was listed in Table 2. *R*^2^ of pseudo-second-order model is much higher than the other, indicating that the pseudo-second order equation is more suitable to describe the CV adsorption with SrCO_3_/g-C_3_N_4_. So the chemisorptions or chemical sorption was the rate-controlling step for the adsorption process.^79^

**Table 2:** Kinetic parameters of different initial CV concentration

*C*_0_ (mg L^−1^)	Pseudo-first-order			Pseudo-second-order			
	*q*_e_ (cal) (mg g^−1^)	*k*_1_ (min^−1^)	*R* ^2^	*q*_e_ (exp) (mg g^−1^)	*q*_e_ (cal) (mg g^−1^)	*k*_2_ (g (mg^−1^ min^−1^)) × 10^−5^	*R* ^2^
1600	16.91	0.014	0.5357	1576.9	1666.7	3.67	0.9425
1800	28.33	0.016	0.7481	1378.6	1428.5	3.53	0.9577
2000	29.53	0.024	0.7822	1143.4	1250.0	8.10	0.9902

### 3.4 Desorption and reuse

In order to evaluate the reusability of SrCO_3_/g-C_3_N_4_, the regeneration process was performed. Adsorption experiment was conducted at the optimal condition as ascribed above in a 250 mL CV solution firstly, after equilibrium the used adsorbent was regenerated by centrifugation and washed several times with methanol and water at room temperature. Finally, the regenerated composite was dried for 24 h at 60 °C for reusing.

**Fig. 13:**
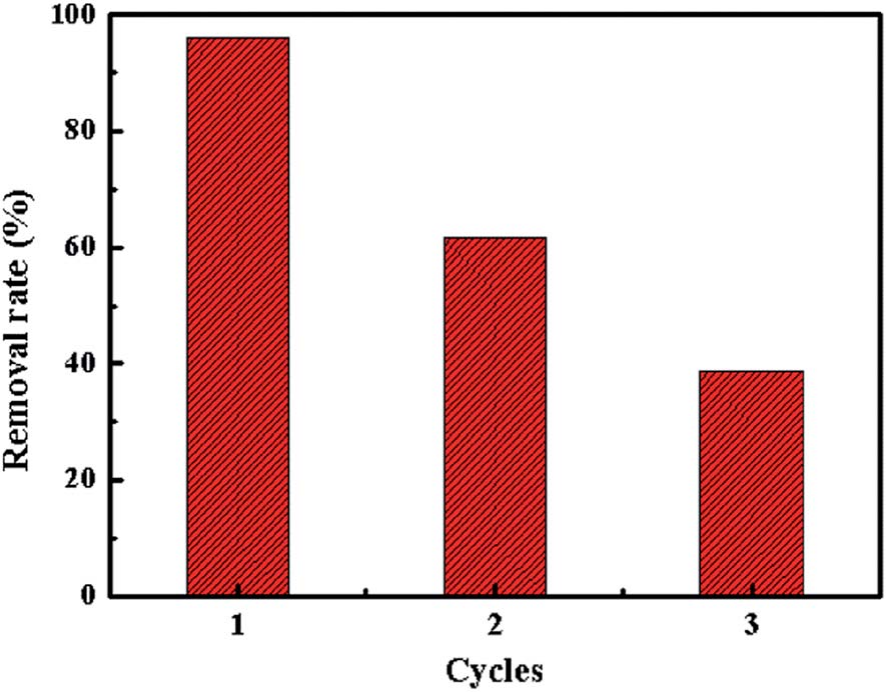
Reusability of the adsorbent.

It could be seen that the removal rate gradually decreased after regeneration in Fig. 13. It might conclude that the adsorption mechanism of this process is not due to the physical adsorption.

### 3.5 Adsorption mechanism of crystal violet

**Fig. 14:**
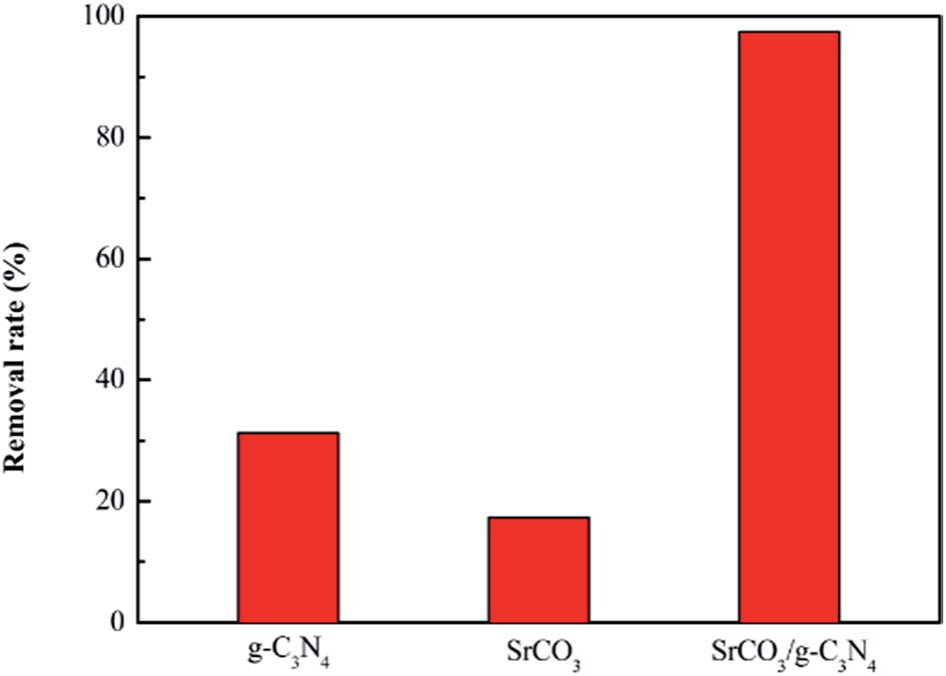
Comparisons of the adsorption effect of the SrCO_3_, g-C_3_N_4_, and SrCO_3_/g-C_3_N_4_.

To further study the adsorption performance of CV, the adsorption experiments were conducted with the calcined SrCO_3_, g-C_3_N_4_ and SrCO_3_/g-C_3_N_4_ firstly. 0.08 g tested materials were dispersed in 80 mL CV solution (10 mg L^−1^), respectively. After 2 h adsorption process, the removal rate of CV for tested materials is exhibited in Fig. 14. It could be seen obviously that the removal ability of g-C_3_N_4_ and SrCO_3_ to CV is much smaller than that of the nanocomposite. The removal rate of g-C_3_N_4_, SrCO_3_ and SrCO_3_/g-C_3_N_4_ were 31.30%, 17.30% and 97.46%, respectively. The adsorption mechanism of g-C_3_N_4_ might be attributed to π–π interaction between the g-C_3_N_4_ and CV, and that for the SrCO_3_, the electrostatic attraction between the carbonate generated from the hydrolytic of SrCO_3_ and the cationic chromogenic groups of CV. And for the SrCO_3_/g-C_3_N_4_, the synergistic effect of π–π interaction and electrostatic attraction highly improved the adsorption properties of the nanocomposite on CV.^50^ According to the above results and the crystal structures, we could deduce that superb adsorption capacity of CV might attribute to the synergistic interaction of the conjugation of g-C_3_N_4_ and the electrostatic attraction of SrCO_3_.

**Fig. 15:**
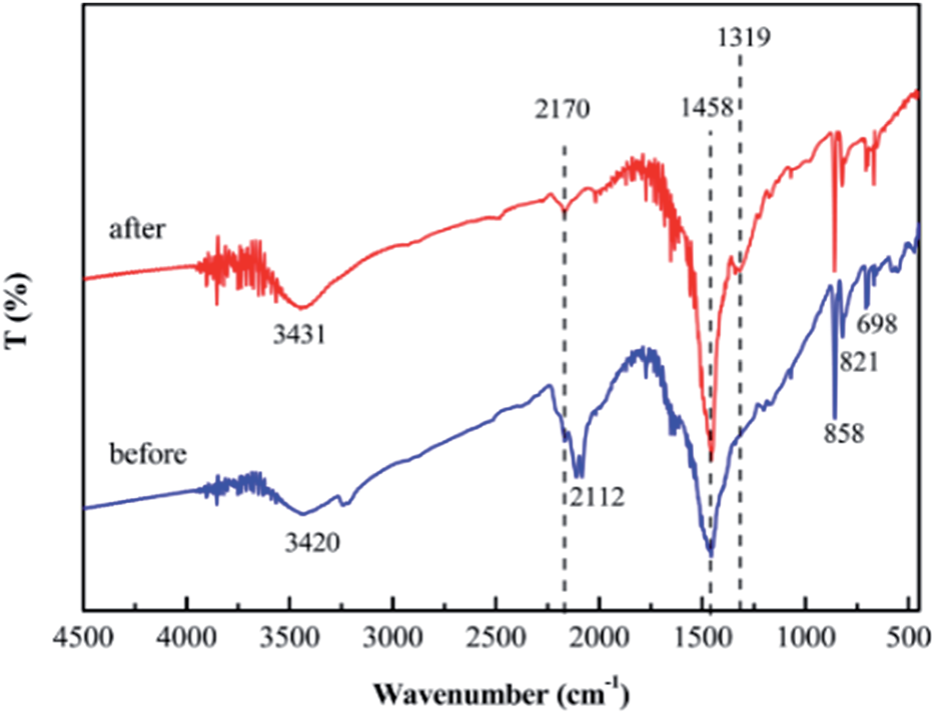
FT-IR spectra of SrCO_3_/g-C_3_N_4_ before and after adsorption.

Furthermore, the FT-IR spectra of SrCO_3_/g-C_3_N_4_ before and after CV adsorption were recorded. As shown in Fig. 15, the peak at 2112 cm^−1^ assigned to the nitrile groups shifted to the 2107 cm^−1^ and the peak at 1319 cm^−1^ assigned to aromatic C–N heterocycles observed clearly. These results indicate that the previously mentioned groups are involved in the adsorption process. Moreover, the intensity of the peak 1458 cm^−1^ corresponding to C–N heterocycle aromatic became stronger, that was for the CV was adsorbed onto the SrCO_3_/g-C_3_N_4_. All the changes of the FT-IR spectra of the SrCO_3_/g-C_3_N_4_ before and after adsorption could indicate that there have been intense interactions between dye molecules and SrCO_3_/g-C_3_N_4_. Furthermore, the result of desorption test might indicate that there has been intense chemical adsorption between the composite and CV. It could deduced that SrCO_3_/g-C_3_N_4_ performed selective adsorption of CV might due to the chemical structure of CV is more symmetrical and conjugation degree is higher than MB and RhB. On the basis of the above analysis, the conjugation and electrostatic interaction would be the driving force for the selective adsorption of CV.

## 4 Conclusions

In summary, a new SrCO_3_/g-C_3_N_4_ composite adsorbent was firstly synthesized with SrCO_3_ and melamine *via* a simple one-step calcination method. The SrCO_3_/g-C_3_N_4_ performed high and specific selective adsorption for CV. The experiment data was well described by the pseudo-second-order model. According to the crystal structural feature of SrCO_3_/g-C_3_N_4_ and the FT-IR analysis of SrCO_3_/g-C_3_N_4_ before and after adsorption of CV, it could be deduced that the synergies of π conjugation and electrostatic interaction would be the mechanism for the selective adsorption of CV. Our findings indicate that g-C_3_N_4_ based composite SrCO_3_/g-C_3_N_4_ could be a promising adsorbent which can potentially be applied for the removal of CV pollutants from aqueous solution.

## Conflicts of interest

There are no conflicts to declare.

## Supplementary Material

## References

[cit1] Zhao Z. W., Sun Y. J., Dong F. (2015). Graphitic carbon nitride based nanocomposites: a review. Nanoscale.

[cit2] Ding F., Yang D., Tong Z. W., Nan Y. H., Wang Y. J., Zou X. Y., Jiang Z. Y. (2017). Graphitic carbon nitride-based nanocomposites as visible-light driven photocatalysts for environmental purification. Environ. Sci.: Nano.

[cit3] Wang X. C., Maeda K., Thomas A., Takanabe K., Xin G., Carlsson J. M., Domen K., Antonietti M. (2009). A metal-free polymeric photocatalyst for hydrogen production from water under visible light. Nat. Mater..

[cit4] Shi A. Y., Li H. H., Yin S., Liu B., Zhang J. C., Wang Y. H. (2017). Effect of conjugation degree and delocalized π-system on the photocatalytic activity of single layer g-C_3_N_4_. Appl. Catal., B.

[cit5] Wu M., Yan J. M., Zhang X. W., Zhao M. (2015). Synthesis of g-C_3_N_4_ with heating acetic acid treated melamine and its photocatalytic activity for hydrogen evolution. Appl. Surf. Sci..

[cit6] Mamba G., Mishra A. K. (2016). Graphitic carbon nitride (g-C_3_N_4_) nanocomposite: A new and exciting generation of visible light driven photocatalysts for environmental pollution remediation. Appl. Catal., B.

[cit7] Zheng Y., Lin L. H., Wang B., Wang X. C. (2015). Graphitic carbon nitride polymers toward sustainable photoredox catalysis. Angew. Chem., Int. Ed..

[cit8] Li J., Yin Y. C., Liu E. Z., Ma Y. N., Wan J., Fan J., Hu X. Y. (2017). *In situ* growing Bi_2_MoO_6_ on g-C_3_N_4_ nanosheets with enhanced photocatalytic hydrogen evolution and disinfection of bacteria under visible light irradiation. J. Hazard. Mater..

[cit9] Katsumata K., Motoyoshi R., Matsushita N., Okada K. (2013). Preparation of graphitic carbon nitride (g-C_3_N_4_)/WO_3_ composites and enhanced visible-light-driven photodegradation of acetaldehyde gas. J. Hazard. Mater..

[cit10] Zhang J. F., Hu Y. F., Jiang X. L., Chen S. F., Meng S. G., Fu X. L. (2014). Design of a direct Z-scheme photocatalyst: Preparation and characterization of Bi_2_O_3_/g-C_3_N_4_ with high visible light activity. J. Hazard. Mater..

[cit11] Masih D., Ma Y. Y., Rohani S. (2017). Graphitic C_3_N_4_ based noble-metal-free photocatalyst systems: A review. Appl. Catal., B.

[cit12] Liu S. W., Chen F., Li S. T., Peng X. X., Xiong Y. (2017). Enhanced photocatalytic conversion of greenhouse gas CO_2_ into solar fuels over g-C_3_N_4_ nanotubes with decorated transparent ZIF-8 nanoclusters. Appl. Catal., B.

[cit13] Wang F., Wang G., Zhang J. C., Li B. Q., Zhang J., Deng J., Chen J. W., Wang R. L. (2017). Novel sulfonated poly (ether ether ketone)/oxidized g-C_3_N_4_ composite membrane for vanadium redox flow battery applications. J. Electroanal. Chem..

[cit14] Mittal A., Mittal J., Malviya A., Kaur D., Gupta V. K. (2010). Adsorption of hazardous dye crystal violet from wastewater by waste materials. J. Colloid Interface Sci..

[cit15] Zhang L. Y., Zhang H. Y., Guo W., Tian Y. (2014). Removal of malachite green and crystal violet cationic dyes from aqueous solution using activated sintering process red mud. Appl. Clay Sci..

[cit16] Pei Y. Y., Wang M., Tian D., Xu X. F., Yuan L. J. (2015). Synthesis of core–shell SiO_2_@MgO with flower like morphology for removal of crystal violet in water. J. Colloid Interface Sci..

[cit17] Sabna V., Thampi S. G., Chandrakaran S. (2016). Adsorption of crystal violet onto functionalized multi-walled carbon nanotubes: Equilibrium and kinetic studies. Ecotoxicol. Environ. Saf..

[cit18] Charkraborty S., Chowdhury S., Saha P. D. (2011). Adsorption of Crystal Violet from aqueous solution onto NaOH-modified rice husk. Carbohydr. Polym..

[cit19] Kumar R., Ahmad R. (2011). Biosorption of hazardous crystal violet dye from aqueous solution onto treated ginger waste (TGW). Desalination.

[cit20] Vinod K. N., Puttaswamy, Ninge Gowda K. N. (2009). Oxidative decolorization of triphenylmethane dyes by chloramine-T in alkaline medium catalyzed by Pd(II): A comparative spectrophotometric kinetic and mechanistic approach. J. Mol. Catal. A: Chem..

[cit21] Yin J. Y., Cai J. J., Yin C., Gao L. F., Zhou J. C. (2016). Degradation performance of crystal violet over CuO@AC and CeO_2_–CuO@AC catalysts using microwave catalytic oxidation degradation method. J. Environ. Chem. Eng..

[cit22] Chen F. T., Fang P. F., Gao Y. P., Liu Z., Liu Y., Dai Y. Q. (2012). Effective removal of high-chroma crystal violet over TiO_2_-based nanosheet by adsorption–photocatalytic degradation. Chem. Eng. J..

[cit23] Jiang Y. R., Lin H. P., Chung W. H., Dai Y. M., Chen C. C. (2015). Controlled hydrothermal synthesis of BiOxCly/BiOmIn composite exhibiting visible-light photocatalytic degradation of crystal violet. J. Hazard. Mater..

[cit24] Popli S., Patel U. D. (2017). Mechanistic aspects of electro-catalytic reduction of Reactive Black 5 dye in a divided cell in the presence of silver nano-particles. Sep. Purif. Technol..

[cit25] Zhang X. Y., Luo H. Q., Li N. B. (2014). Crystal violet as an i-motif structure probe for reversible and label-free pH-driven electrochemical switch. Anal. Biochem..

[cit26] Khataee A., Gholami P., Vahid B., Joo S. W. (2016). Heterogeneous sono-Fenton process using pyrite nanorods prepared by non-thermal plasma for degradation of an anthraquinone dye. Ultrason. Sonochem..

[cit27] Jiang B., Zheng J. T., Liu Q., Wu M. B. (2012). Degradation of azo dye using non-thermal plasma advanced oxidation process in a circulatory airtight reactor system. Chem. Eng. J..

[cit28] Chen J. Y., Du Y. L., Shen Z. J., Lu S. S., Su K. Z., Yuan S. J., Hu Z. H., Zhang A. Y., Feng J. W. (2017). Non-thermal plasma and BiPO_4_ induced degradation of aqueous crystal violet. Sep. Purif. Technol..

[cit29] Nair V., Panigrahy A., Vinu R. (2014). Development of novel chitosan–lignin composite for adsorption of dyes and metal ions from wastewater. Chem. Eng. J..

[cit30] Yang S. X., Wang L. Y., Zhang X. D., Yang W. J., Song G. L. (2015). Enhanced adsorption of Congo red dye by functionalized carbon nanotube/mixed metal oxides nanocomposite derived from layer doubled hydroxide precursor. Chem. Eng. J..

[cit31] Njoku V. O., Foo K. Y., Asif M., Hameed B. H. (2014). Preparation of activated carbons from rambutan (Nepheliumlappaceum) peel by microwave-induced KOH activation for acid yellow 17 dye adsorption. Chem. Eng. J..

[cit32] Liu X. X., Gong W. P., Luo J., Zou C. T., Yang Y., Yang S. J. (2016). Selective adsorption of cationic dyes from aqueous solution by polyoxometalate-based metal-organic framework composite. Appl. Surf. Sci..

[cit33] Faradi S., Amini M. M., Dusek M., Kucerakova M., Mahmoudi F. (2017). A new nanohybrid material constructed from Keggin-type polyoxometalate and Cd(II) semicarbazone
Schiff base complex with excellent adsorption properties for the removal of cationic dye pollutants. J. Mol. Struct..

[cit34] Jia Z. G., Li Z. Y., Ni T., Li S. B. (2017). Adsorption of low-cost absorption materials based on biomass (Cortaderiaselloana flower spikes) for dye removal: Kinetics, isotherms and thermodynamic studies. J. Mol. Liq..

[cit35] Bhattacharyya A., Mondal D., Roy I., Sarkar G., Saha N. R., Rana D., Ghosh T. K., Mandal D., Chakraborty M., Chattopadhyay D. (2017). Studies of the kinetics and mechanism of the removal process of proflavine dye through adsorption by grapheme oxide. J. Mol. Liq..

[cit36] Regti A., Laamari M. R., Stiriba S. E., Haddad M. E. (2017). Use of response factorial design for process optimization of basic dye adsorption onto activated carbon derived from Persea species. Microchem. J..

[cit37] Carrales-Alvarado D. H., Ocampo-Perez R., Leyva-Ramos R., Rivera-Utrilla J. (2014). Removal of the antibiotic metronidazole by adsorption on various carbon materials from aqueous phase. J. Colloid Interface Sci..

[cit38] Li H., An N. H., Liu G., Li J. L., Liu N., Jia M. J., Zhang W. X., Yuan X. L. (2015). Adsorption behaviors of methyl orange dye on nitrogen-doped mesoporous carbon materials. J. Colloid Interface Sci..

[cit39] Zhang L. Y., Zhang H. Y., Guo W., Tian Y. L. (2014). Removal of malachite green and crystal violet cationic dyes from aqueous solution using activated sintering process red mud. Appl. Clay Sci..

[cit40] Sarma G. K., Gupta S. S., Bhattacharyya K. G. (2016). Adsorption of Crystal violet on raw and acid-treated montmorillonite, K10, in aqueous suspension. J. Environ. Manage..

[cit41] Gupta V. K., Agarwal S., Olgun A., Demir H., Yola M. L., Atar N. (2016). Adsorptive properties of molasses modified boron enrichment waste based nanoclay for removal of basic dyes. J. Ind. Eng. Chem..

[cit42] Zhu R. L., Chen Q. Z., Liu H. Y., Ge F., Zhu L. F., Zhu J. X., He H. P. (2014). Montmorillonite as a multifunctional adsorbent can simultaneously remove crystal violet, cetyltrimethylammonium, and 2-naphthol from water. Appl. Clay Sci..

[cit43] Jia Z. G., Li Z. Y., Ni T., Li S. B. (2017). Adsorption of low-cost absorption materials based on biomass (Cortaderiaselloana flower spikes) for dye removal: Kinetics, isotherms and thermodynamic studies. J. Mol. Liq..

[cit44] Shoukat S., Bhatti H. N., Iqbal M., Noreen S. (2017). Mango stone biocomposite preparation and application for crystal violet adsorption: A mechanistic study. Microporous Mesoporous Mater..

[cit45] Sun P. F., Hui C., Wang S., Wan L., Zhao Y. H. (2016). Bacillus amyloliquefaciens biofilm as a novel biosorbent for the removal of crystal violet from solution. Colloids Surf., B.

[cit46] Lam S. S., Liew R. K., Wong Y. M., Yek P. N. Y., Ma N. L., Lee C. L., Chase H. A. (2017). Microwave-assisted pyrolysis with chemical activation, an innovative method to convert orange peel into activated carbon with improved properties as dye adsorbent. J. Cleaner Prod..

[cit47] Lakovleva E., Sillanpaä M., Maydannik P., Liu J. T., Allen S., Albadarin A. B., Mangwandi C. (2017). Manufacturing of novel low-cost adsorbent: Co-granulation of limestone and coffee waste. J. Environ. Manage..

[cit48] Chicinaş R. P., Bedelean H., Stefan R., Măicăneanu A. (2018). Ability of a montmorillonitic clay to interact with cationic and anionic dyes in aqueous solutions. J. Mol. Struct..

[cit49] Thue P. S., Sophia A. C., Lima E. C., Wamba A. G. N., de Alencar W. S., dos Reis G. S., Rodembusch F. S. (2018). Synthesis and characterization of a novel organic-inorganic hybrid clay adsorbent for the removal of acid red 1 and acid green 25 from aqueous solutions. J. Cleaner Prod..

[cit50] Zheng Y., Chen D. Y., Li N. J., Xu Q. F., Li H., He J. H., Lu J. M. (2017). Highly efficient simultaneous adsorption and biodegradation of a highly-concentrated anionic dye by a high-surface-area carbon-based biocomposite. Chemosphere.

[cit51] Tahir N., Bhatti H. N., Lqbal M., Noreen S. (2017). Biopolymers composites with peanut hull waste biomass and application for Crystal Violet adsorption. Int. J. Biol. Macromol..

[cit52] Shoukat S., Bhatti H. N., Lqbal M., Noreen S. (2017). Mango stone biocomposite preparation and application for crystal violet adsorption: A mechanistic study. Microporous Mesoporous Mater..

[cit53] Tahir N., Bhatti H. N., Iqbal M., Noreen S. (2017). Biopolymers composites with peanut hull waste biomass and application for Crystal Violet adsorption. Int. J. Biol. Macromol..

[cit54] Cai X. G., He J. Y., Chen L., Li Y. L., Zhang K. S., Jin Z., Liu J. Y., Wang C. M., Wang X. G., Kong L. T. (2017). A 2D-g-C_3_N_4_ nanosheet as an eco-friendly adsorbent for various environmental pollutants in water. Chemosphere.

[cit55] Ding X. R., Zhu J., Zhang Y., Xia Q., Bi W. T., Yang X. D., Yang J. F. (2016). Separation and concentration of natural products by fast forced adsorption using well-dispersed velvet-like graphitic carbon nitride with response surface methodology optimization. Talanta.

[cit56] Bao N., Hu X. D., Zhang Q. Z., Miao X. H., Jie X. Y., Zhou S. (2017). Synthesis of porous carbon-doped g-C_3_N_4_ nanosheets with enhanced visible-light photocatalytic activity. Appl. Surf. Sci..

[cit57] Mamba G., Mishra A. K. (2016). Graphitic carbon nitride (g-C_3_N_4_) nanocomposites: A new and exciting generation of visible light driven photocatalysts for environmental pollution remediation. Appl. Catal., B.

[cit58] Cao S. W., Low J. X., Yu J. G., Jaroniec M. (2015). Polymeric photocatalysts based on graphitic carbon nitride. Adv. Mater..

[cit59] Tan G. Q., She L. N., Liu T., Xu C., Ren H. J., Xia A. (2017). Ultrasonic chemical synthesis of hybrid mpg-C_3_N_4_/BiPO_4_ heterostructured photocatalysts with improved visible light photocatalytic activity. Appl. Catal., B.

[cit60] Liu C. Y., Zhang Y. H., Dong F., Reshak A. H., Ye L. Q., Pinna N., Zeng C., Zhang T. R., Huang H. W. (2017). Chlorine intercalation in graphitic carbon nitride for efficient photocatalysis. Appl. Catal., B.

[cit61] He X. B., Yin F. X., Chen J. N., Ye C. Y. (2017). Co-SrCO_3_/N-doped carbon: a highly efficient hybrid electrocatalyst for the oxygen reduction reaction and Zn–air batteries. Inorg. Chem. Front..

[cit62] Zhu W. C., Zhang G. L., Li J., Zhang Q., Piao X. L., Zhu S. L. (2010). Hierarchical mesoporous SrCO_3_ submicron spheres derived from reaction-limited aggregation induced “rod-to-dumbbell-to-sphere” self-assembly. CrystEngComm.

[cit63] Du J. M., Liu Z. M., Li Z. H., Han B. X., Huang Y., Zhang J. L. (2005). Synthesis of mesoporous SrCO_3_ spheres and hollow CaCO_3_ spheres in room-temperature ionic liquid. Microporous Mesoporous Mater..

[cit64] Zhu W. C., Liang Z. Z., Liu X. F., Zhang H., Zheng Y. J., Piao X. L., Zhang Q. (2012). Soft-template self-assembly of hierarchical mesoporous SrCO_3_ by low-temperature hydrothermal route and their application as adsorbents for methylene blue and heavy metal ions. Powder Technol..

[cit65] Wu S. S., Yin S. F., Cao H. Q., Lu Y. X., Yin J. F., Li B. J. (2011). Glucosan controlled biomineralization of SrCO_3_ complex nanostructures with superhydrophobicity and adsorption properties. J. Mater. Chem..

[cit66] Bai X., Wang L., Zong R., Zhu Y. (2013). Photocatalytic activity enhanced *via* g-C_3_N_4_ nanoplates to nanorods. J. Phys. Chem..

[cit67] Xing W. N., Li C. M., Chen G., Han Z. H., Zhou Y. S., Hu Y. D., Meng Q. Q. (2017). Incorporating a novel metal-free interlayer into g-C_3_N_4_ frame work for efficiency enhanced photocatalytic H_2_ evolution activity. Appl. Catal., B.

[cit68] Ni S. B., Yang W. L., Li T. (2011). Hydrothermal synthesis and photoluminescence properties of SrCO_3_. Mater. Lett..

[cit69] Tahmasian A., Safarifard V., Morsali A., Joo S. W. (2014). Sonochemical syntheses of a new fibrous-like nano-scale strontium(II) 3D coordination polymer; precursor for the fabrication of a strontium carbonate nanostructure. Polyhedron.

[cit70] Huang Z. F., Song J. J., Pan L., Wang Z. M., Zhang X. Q., Zou J. J., Mi W. B., Zhang X. W., Wang L. (2015). Carbon nitride with simultaneous porous network and O-doping for efficient solar-energy-driven hydrogen evolution. Nano Energy.

[cit71] Jo W. K., Selvam C. S. (2015). Enhanced visible light-driven photocatalytic performance of ZnO-g-C_3_N_4_ coupled with graphene oxide as a novel ternary nanocomposite. J. Hazard. Mater..

[cit72] Chen D. M., Yang J. J., Ding H. (2017). Synthesis of nanoporous carbon nitride using calcium carbonate as templates with enhanced visible-light photocatalytic activity. Appl. Surf. Sci..

[cit73] Papailias I., Giannakopoulou T., Todorova N., Demotukali D., Vaimakis T., Trapalis C. (2015). Effect of processing temperature on structure and photocatalytic properties of g-C_3_N_4_. Appl. Surf. Sci..

[cit74] Zhang Y. X., Wu J., Deng Y. Y., Xin Y. J., Liu H. L., Ma D., Bo N. (2017). Synthesis and visible-light photocatalytic property of Ag/GO/g-C_3_N_4_ ternary composite. J. Mater. Sci. Eng. B.

[cit75] Wu M., Yan J. M., Tang X. N., Zhao M., Jiang Q. (2014). Synthesis of potassium-modified graphitic carbon nitride with high photocatalytic activity for hydrogen evolution. ChemSusChem.

[cit76] Wu M., Yan J. M., Zhang X. W., Zhao M. (2015). Synthesis of g-C_3_N_4_ with heating acetic acid treated melamine and its photocatalytic activity for hydrogen evolution. Appl. Surf. Sci..

[cit77] Lv J. L., Dai K., Zhang J. F., Liu Q., Liang C. H., Zhu G. P. (2017). Facile constructing novel 2D porous g-C_3_N_4_/BiOBr hybrid with enhanced visible-light-driven photocatalytic activity. Sep. Purif. Technol..

[cit78] Álvarez-Torrellas S., Ribeiro R. S., Gomes H. T., Ovejero G., García J. (2016). Removal of antibiotic compounds by adsorption using glycerol-based carbon materials. Chem. Eng. J..

[cit79] Ho Y. S., McKay G. (1999). Pseudo-second order model for sorption processes. Process Biochem..

